# The genome sequence of the European Snow Vole, *Chionomys nivalis *(Martins, 1842)

**DOI:** 10.12688/wellcomeopenres.23922.1

**Published:** 2025-03-20

**Authors:** Franc Janžekovič, Boris Kryštufek, Wellcome Sanger Institute Tree of Life Management, Samples and Laboratory team, Wellcome Sanger Institute Scientific Operations: Sequencing Operations, Wellcome Sanger Institute Tree of Life Core Informatics team, Tree of Life Core Informatics collective

**Affiliations:** 1 Faculty of Natural Sciences and Mathematics, University of Maribor, Maribor, Slovenia; 2 Slovenian Museum of Natural History, Ljubljana, Slovenia

**Keywords:** Chionomys nivalis, European Snow Vole, genome sequence, chromosomal, Rodentia

## Abstract

We present a genome assembly from a male specimen of *Chionomys nivalis* (European Snow Vole; Chordata; Mammalia; Rodentia; Cricetidae). The genome sequence has a total length of 2,393.39 megabases. Most of the assembly (98.05%) is scaffolded into 28 chromosomal pseudomolecules, including the X and Y sex chromosomes. The mitochondrial genome has also been assembled and is 16.29 kilobases in length.

## Species taxonomy

Eukaryota; Opisthokonta; Metazoa; Eumetazoa; Bilateria; Deuterostomia; Chordata; Craniata; Vertebrata; Gnathostomata; Teleostomi; Euteleostomi; Sarcopterygii; Dipnotetrapodomorpha; Tetrapoda; Amniota; Mammalia; Theria; Eutheria; Boreoeutheria; Euarchontoglires; Glires; Rodentia; Myomorpha; Muroidea; Cricetidae; Arvicolinae; *Chionomys*; *Chionomys nivalis* (Martins, 1842) (NCBI:txid269649)

## Background

**Figure 1.**
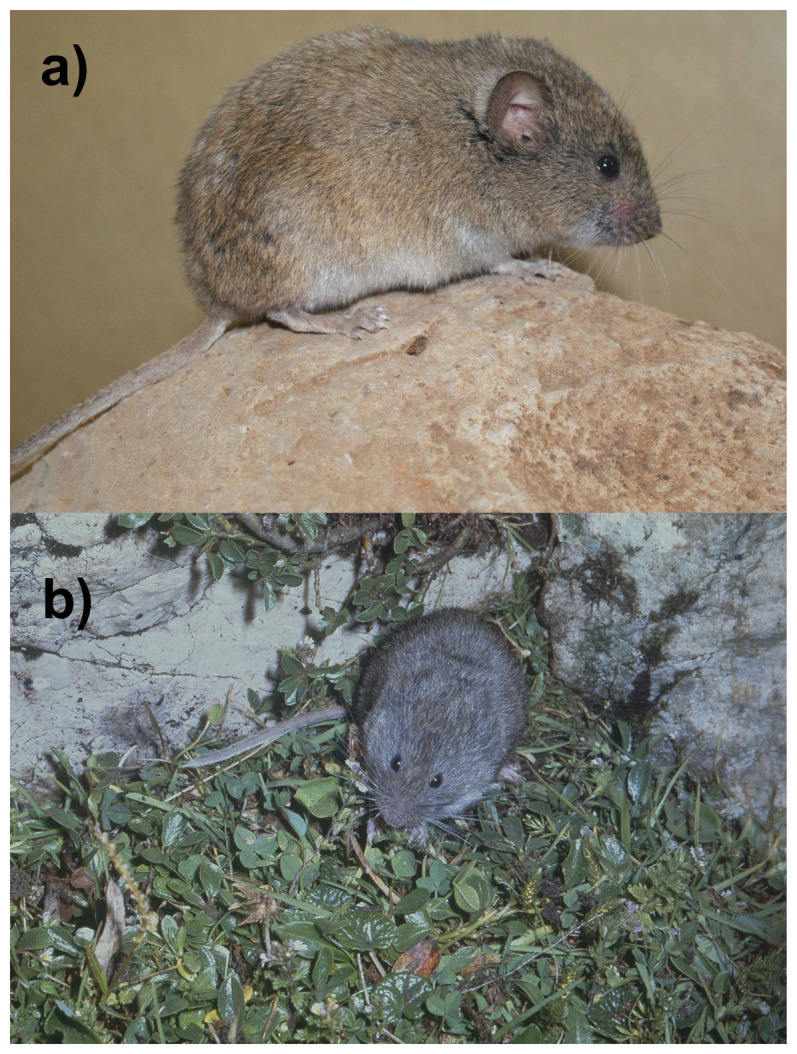
Photographs of *Chionomys nivalis* (not the specimen used for genome sequencing). **a**) Photo (CHINIV_AK). *Chionomys nivalis*, Syria. Photo Alenka Kryštufek. **b**) Photo (Chionomys_nivalis_JC). *Chionomys nivalis*, Slovenia. Photo Jaroslav Červeny.

The European snow vole ( *Chionomys nivalis*) ( Figure 1) is a rodent from the speciose family Cricetidae. It is characterised by a comparatively large size (body mass is 30–78 g), long tail, large ears and long whiskers. The fur is long, soft, and dense. The colour varies among populations, but shades of grey are always present. The belly is paler, and the tail and paws are frequently whitish ( Kryštufek & Shenbrot, 2022; Pardiñas *et al.*, 2017).

The distribution range of *C. nivalis* covers mountainous regions from southwestern Europe, through the Pyrenees, Alps and Carpathians, the mountain ranges of the Italian and Balkan Peninsulas, as far east as the Middle East, including Anatolia, the Caucasus, mountains of Iran and Kopetdag. Snow voles seek shelter in deep crevices and fissures, and they depend on fractured rocky substrate. They have been recorded from close to sea level to over 5,000 m in elevation, however, they are most abundant in the mountains above the tree line. Their range is fragmented at various spatial scales, and many local populations are small and isolated. A significant part of their diurnal activity occurs during the daytime, so these voles are frequently observed by mountaineers. Home ranges of males encompass several females which have smaller and non-overlapping ranges.

The diet of *C. nivalis* is strictly herbivorous and daily consumption consists of 5–10 g of dry matter. These voles typically reproduce in spring and summer, the length of the breeding season becoming shorter at higher elevations. Females produce two to three litters per season, each with two to six young. The gestation period is 20 to 22 days, and the young are born nude, blind and helpless. They start leaving the nest when two to three weeks old and become independent at about one month of age. Snow voles are preyed upon by owls and weasels ( Kryštufek & Shenbrot, 2022; Pardiñas *et al.*, 2017). The species is classified as “Least Concern” on the IUCN Red List, indicating that it is not at immediate risk of extinction ( Kryštufek, 2016).

We present a chromosome-level genome sequence, produced as part of the European Reference Genome Atlas (ERGA) Pilot Project ( Mc Cartney *et al.*, 2024).

## Genome sequence report

### Sequencing data

The genome of a specimen of *Chionomys nivalis* as sequenced using Pacific Biosciences single-molecule HiFi long reads, generating 64.28 Gb (gigabases) from 6.05 million reads. GenomeScope analysis of the PacBio HiFi data estimated the haploid genome size at 2,360.44 Mb, with a heterozygosity of 0.48% and repeat content of 17.15%. These values provide an initial assessment of genome complexity and the challenges anticipated during assembly. Based on this estimated genome size, the sequencing data provided approximately 26.0x coverage of the genome. Chromosome conformation Hi-C data produced 519.19 Gb from 3,438.34 million reads. Table 1 summarises the specimen and sequencing information.

**Table 1.** Specimen and sequencing data for *Chionomys nivalis*.

Project information			
**Study title**	Chionomys nivalis (European snow vole)		
**Umbrella BioProject**	PRJEB59810		
**Species**	*Chionomys nivalis*		
**BioSpecimen**	SAMEA13217622		
**NCBI taxonomy ID**	269649		
Specimen information			
**Technology**	**ToLID**	**BioSample accession**	**Organism part**
**PacBio long read ** **sequencing**	mChiNiv1	SAMEA13217627	muscle
**Hi-C sequencing**	mChiNiv1	SAMEA13217627	muscle
**RNA sequencing**	mChiNiv1	SAMEA13217623	brain
Sequencing information			
**Platform**	**Run accession**	**Read count**	**Base count (Gb)**
**Hi-C Illumina NovaSeq ** **6000**	ERR10890757	3.44e+09	519.19
**PacBio Sequel IIe**	ERR10879946	1.79e+06	21.39
**PacBio Sequel IIe**	ERR10879944	2.13e+06	19.51
**PacBio Sequel IIe**	ERR10879945	2.12e+06	23.38
**RNA Illumina NovaSeq ** **6000**	ERR10908618	6.35e+07	9.59

### Assembly statistics

The primary haplotype was assembled, and contigs corresponding to an alternate haplotype were also deposited in INSDC databases. The assembly was improved by manual curation, which corrected 41 misjoins or missing joins and removed two haplotypic duplications. These interventions decreased the scaffold count by 15.71%, and increased the scaffold N50 by 4.81%. The final assembly has a total length of 2,393.39 Mb in 160 scaffolds, with 197 gaps, and a scaffold N50 of 88.84 Mb ( Table 2).

**Table 2.** Genome assembly data for *Chionomys nivalis*.

Genome assembly		
Assembly name	mChiNiv1.1	
Assembly accession	GCA_950005125.1	
*Alternate haplotype accession*	*GCA_950005115.1*	
Assembly level for primary assembly	chromosome	
Span (Mb)	2,393.39	
Number of contigs	357	
Number of scaffolds	160	
Longest scaffold (Mb)	205.9	
Assembly metric	Measure	*Benchmark*
Contig N50 length	30.96 Mb	*≥ 1 Mb*
Scaffold N50 length	88.84 Mb	*= chromosome N50*
Consensus quality (QV)	Primary: 60.4; alternate: 60.4; combined: 60.4	*≥ 40*
*k*-mer completeness	Primary: 91.06%; alternate: 78.64%; combined: 99.40%	*≥ 95%*
BUSCO *	C:96.2%[S:93.3%,D:2.9%], F:0.6%,M:3.2%,n:13,798	*S > 90%; D < 5%*
Percentage of assembly mapped to chromosomes	98.05%	*≥ 90%*
Sex chromosomes	X and Y	*localised homologous * *pairs*
Organelles	Mitochondrial genome: 16.29 kb	*complete single alleles*

* BUSCO scores based on the glires_odb10 BUSCO set using version 5.3.2. C = complete [S = single copy, D = duplicated], F = fragmented, M = missing, n = number of orthologues in comparison.

**Figure 2.**
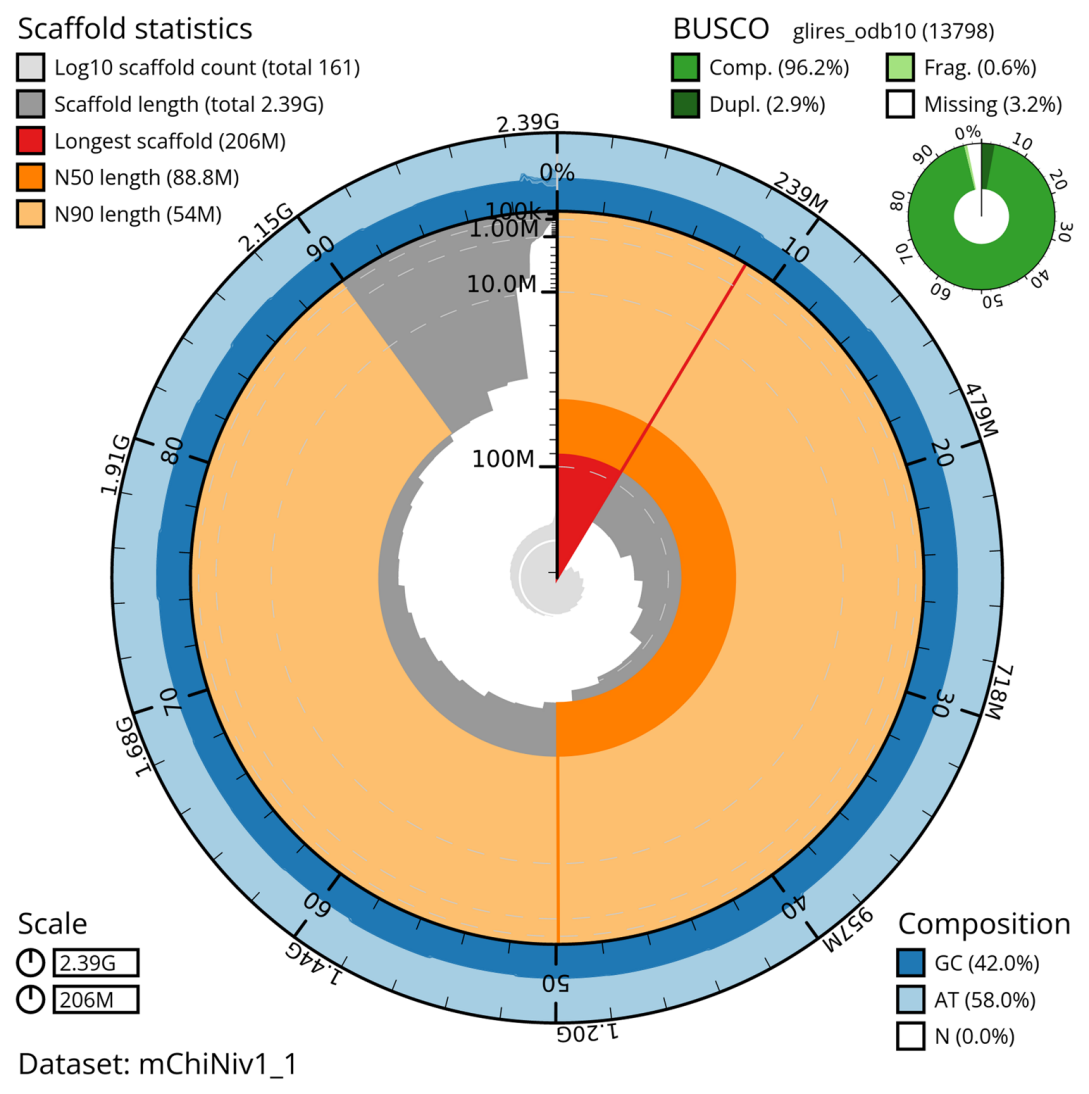
Genome assembly of *Chionomys nivalis*, mChiNiv1.1: metrics. The BlobToolKit snail plot provides an overview of assembly metrics and BUSCO gene completeness. The circumference represents the length of the whole genome sequence, and the main plot is divided into 1,000 bins around the circumference. The outermost blue tracks display the distribution of GC, AT, and N percentages across the bins. Scaffolds are arranged clockwise from longest to shortest and are depicted in dark grey. The longest scaffold is indicated by the red arc, and the deeper orange and pale orange arcs represent the N50 and N90 lengths. A light grey spiral at the centre shows the cumulative scaffold count on a logarithmic scale. A summary of complete, fragmented, duplicated, and missing BUSCO genes in the glires_odb10 set is presented at the top right. An interactive version of this figure is available at https://blobtoolkit.genomehubs.org/view/mChiNiv1_1/dataset/mChiNiv1_1/snail.

**Figure 3.**
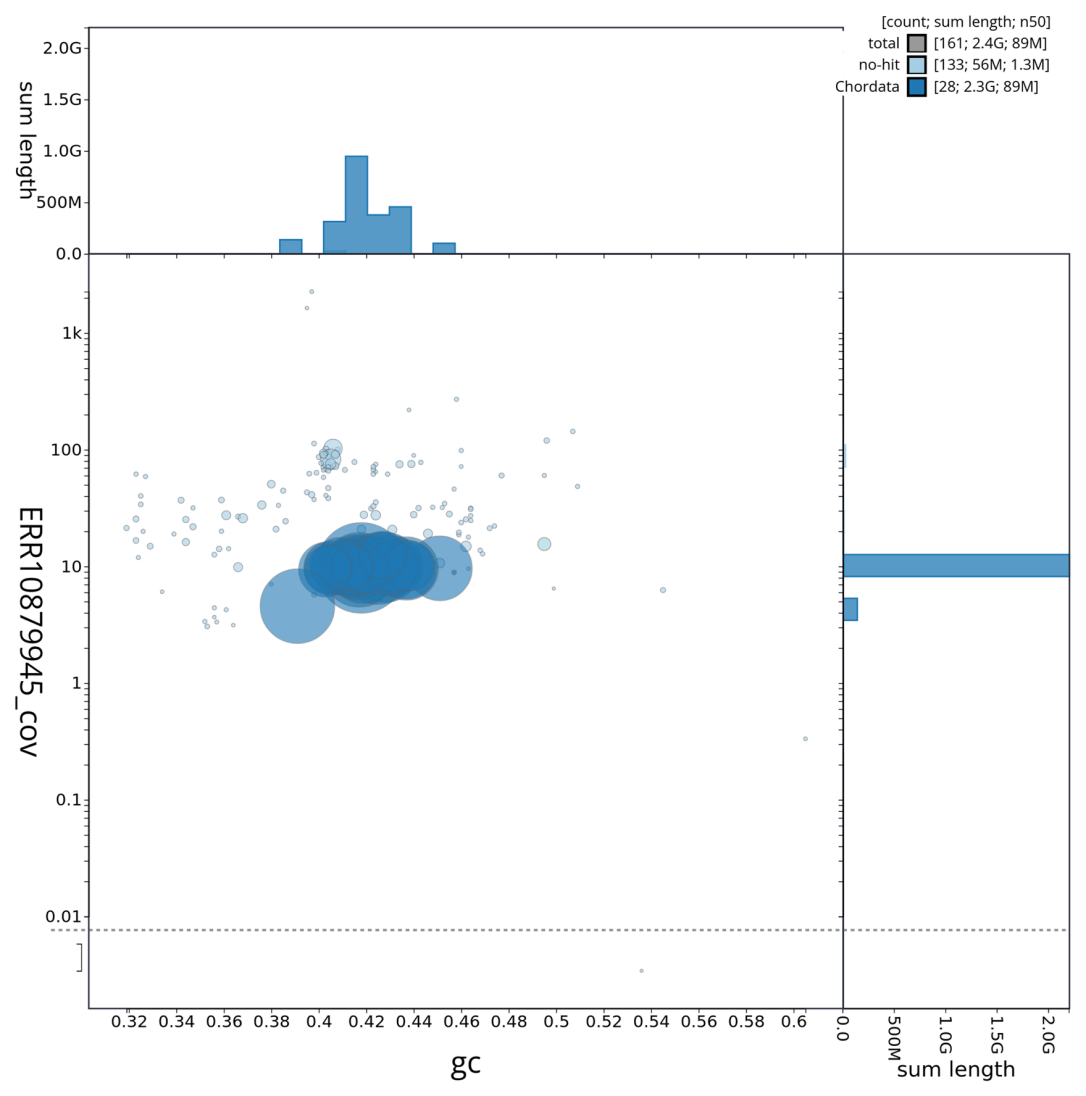
Genome assembly of *Chionomys nivalis*, mChiNiv1.1: BlobToolKit GC-coverage plot. Sequences are coloured by phylum. Circles are sized in proportion to sequence length. Histograms show the distribution of sequence length sum along each axis. An interactive version of this figure is available at https://blobtoolkit.genomehubs.org/view/mChiNiv1_1/dataset/mChiNiv1_1/blob.

**Figure 4.**
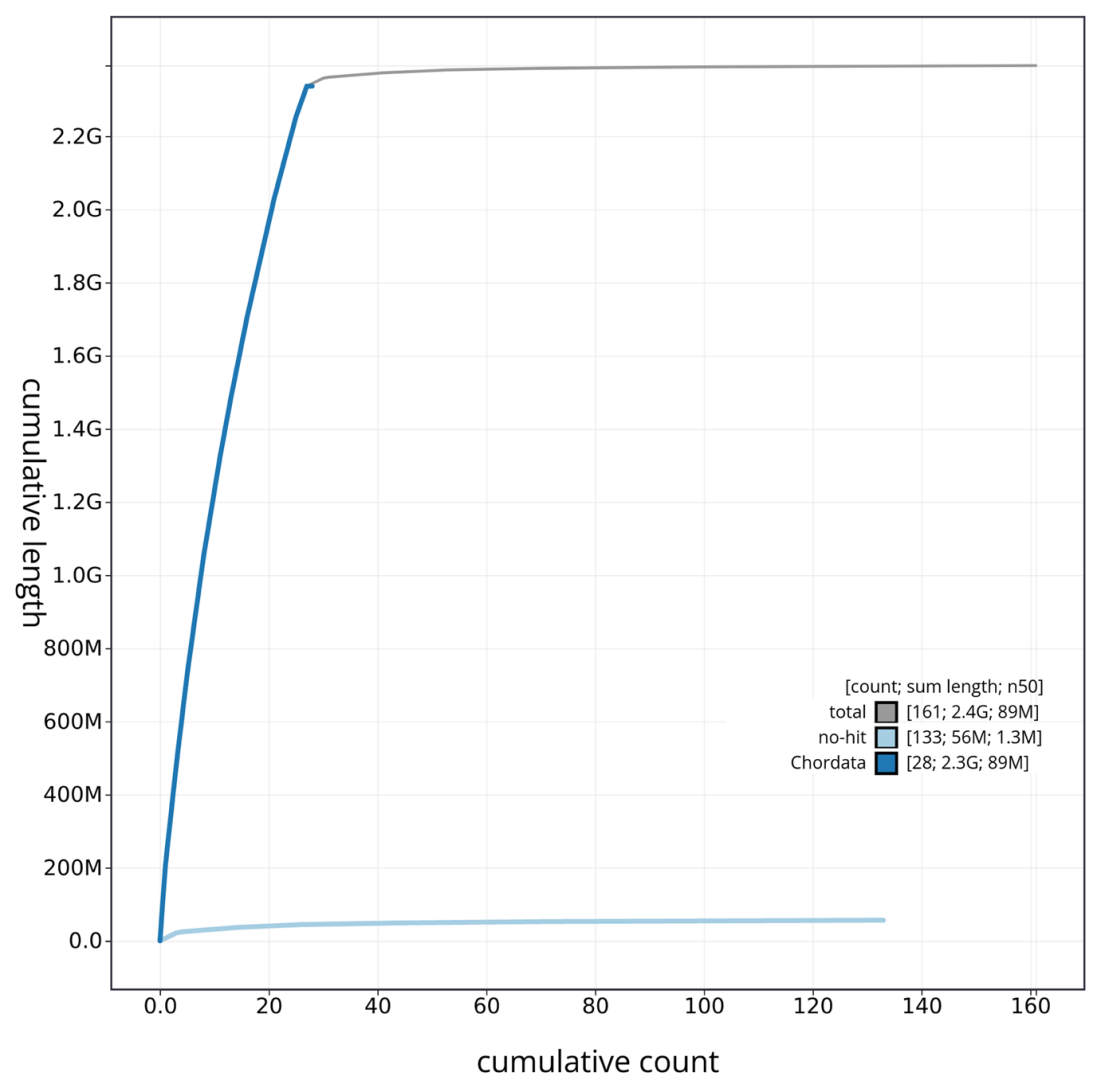
Genome assembly of *Chionomys nivalis* mChiNiv1.1: BlobToolKit cumulative sequence plot. The grey line shows cumulative length for all sequences. Coloured lines show cumulative lengths of sequences assigned to each phylum using the buscogenes taxrule. An interactive version of this figure is available at https://blobtoolkit.genomehubs.org/view/mChiNiv1_1/dataset/mChiNiv1_1/cumulative.

The snail plot in Figure 2 provides a summary of the assembly statistics, indicating the distribution of scaffold lengths and other assembly metrics. Figure 3 shows the distribution of scaffolds by GC proportion and coverage. Figure 4 presents a cumulative assembly plot, with separate curves representing different scaffold subsets assigned to various phyla, illustrating the completeness of the assembly.

**Figure 5.**
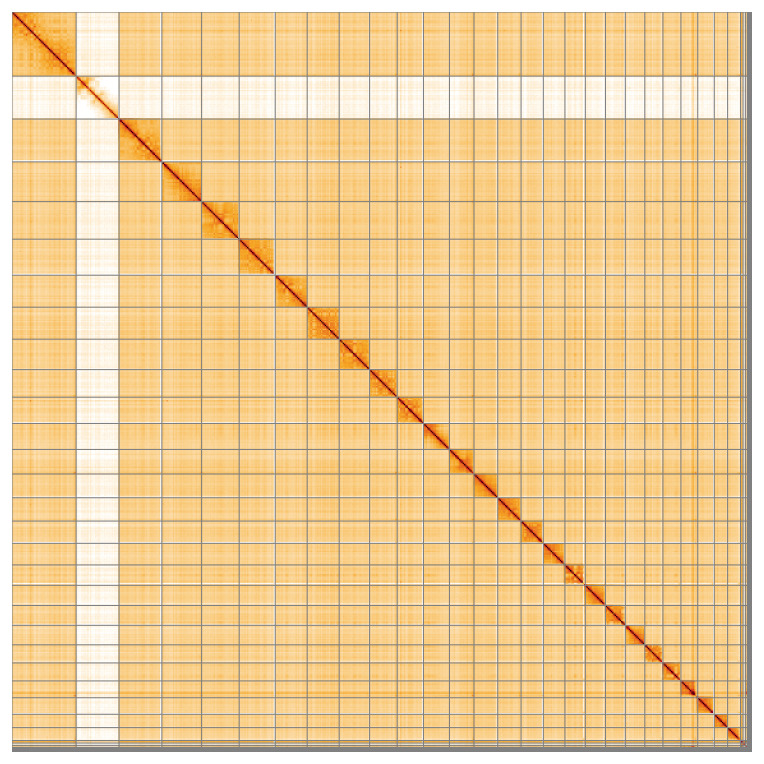
Genome assembly of *Chionomys nivalis*: Hi-C contact map of the mChiNiv1.1 assembly, visualised using HiGlass. Chromosomes are shown in order of size from left to right and top to bottom. An interactive version of this figure may be viewed at https://genome-note-higlass.tol.sanger.ac.uk/l/?d=dYKVod2uRnCGPjHf_tQVUw.

Most of the assembly sequence (97.94%) was assigned to 28 chromosomal-level scaffolds, representing 26 autosomes and the X and Y sex chromosomes. These chromosome-level scaffolds, confirmed by Hi-C data, are named according to size ( Figure 5; Table 3).

**Table 3.** Chromosomal pseudomolecules in the genome assembly of *Chionomys nivalis*, mChiNiv1.

INSDC accession	Name	Length (Mb)	GC%
OX465448.1	1	205.9	42
OX465450.1	2	137.24	41.5
OX465451.1	3	127.61	42.5
OX465452.1	4	120.22	43
OX465453.1	5	115.47	42
OX465454.1	6	102.99	41.5
OX465455.1	7	102.4	45
OX465456.1	8	97.49	43.5
OX465457.1	9	88.84	43
OX465458.1	10	84.11	41.5
OX465459.1	11	83.25	43.5
OX465460.1	12	78.63	41
OX465461.1	13	76.51	42
OX465462.1	14	74.49	41.5
OX465463.1	15	71.44	40.5
OX465464.1	16	69.32	40.5
OX465465.1	17	65.54	43
OX465466.1	18	64.6	41.5
OX465467.1	19	63.9	43
OX465468.1	20	62.3	41
OX465469.1	21	57.93	44
OX465470.1	22	57.73	42
OX465471.1	23	54.0	42.5
OX465472.1	24	53.27	40.5
OX465473.1	25	42.74	41.5
OX465474.1	26	41.32	40.5
OX465449.1	X	137.63	39
OX465475.1	Y	7.2	40.5
OX465476.1	MT	0.02	39.5

The mitochondrial genome was also assembled. This sequence is included as a contig in the multifasta file of the genome submission and as a standalone record in GenBank.

### Assembly quality metrics

The estimated Quality Value (QV) and *k*-mer completeness metrics, along with BUSCO completeness scores, were calculated for each haplotype and the combined assembly. The QV reflects the base-level accuracy of the assembly, while *k*-mer completeness indicates the proportion of expected *k*-mers identified in the assembly. BUSCO scores provide a measure of completeness based on benchmarking universal single-copy orthologues.

The primary haplotype has a QV of 60.4, and the combined primary and alternate assemblies achieve an estimated QV of 60.4. The *k*-mer completeness for the primary haplotype is 91.06%, and for the alternate haplotype it is 78.64%. The combined primary and alternate assemblies achieve a *k*-mer completeness of 99.40%. BUSCO v.5.3.2 analysis using the glires_odb10 reference set ( *n* = 13,798) indicated a completeness score of 96.2% (single = 93.3%, duplicated = 2.9%). A full set of BUSCO scores is available at https://blobtoolkit.genomehubs.org/view/mChiNiv1_1/dataset/mChiNiv1_1/busco.

Table 2 provides assembly metric benchmarks adapted from Rhie *et al.* (2021) and the Earth BioGenome Project (EBP) Report on Assembly Standards September 2024. The assembly achieves the EBP reference standard of **7.C.Q60**.

## Methods

### Sample acquisition

An adult male *Chionomys nivalis* (specimen ID ERGA FJ SI 01, ToLID mChiNiv1) was collected from Vršič (latitude 46.4722, longitude 13.7266) on 2021-07-15. The animal was euthanized using ether in a chamber. Tissue samples (brain, kidney, liver, muscle, testis) were snap-frozen immediately after harvesting and stored at –80 °C. The specimen was collected and identified by Franc Janžekovič and Boris Kryštufek (University of Maribor, Faculty of Natural Science and Mathematics and Slovenian Museum of Natural History).

### Nucleic acid extraction

The workflow for high molecular weight (HMW) DNA extraction at the Wellcome Sanger Institute (WSI) Tree of Life Core Laboratory includes a sequence of procedures: sample preparation and homogenisation, DNA extraction, fragmentation and purification. Detailed protocols are available on protocols.io ( Denton *et al.*, 2023b). The mChiNiv1 sample was prepared for DNA extraction by weighing and dissecting it on dry ice ( Jay *et al.*, 2023). Tissue from the muscle was homogenised using a PowerMasher II tissue disruptor ( Denton *et al.*, 2023a). HMW DNA was extracted using the Automated MagAttract v1 protocol ( Sheerin *et al.*, 2023). DNA was sheared into an average fragment size of 12–20 kb in a Megaruptor 3 system ( Todorovic *et al.*, 2023). Sheared DNA was purified by solid-phase reversible immobilisation, using AMPure PB beads to eliminate shorter fragments and concentrate the DNA ( Strickland *et al.*, 2023). The concentration of the sheared and purified DNA was assessed using a Nanodrop spectrophotometer and a Qubit Fluorometer using the Qubit dsDNA High Sensitivity Assay kit. The fragment size distribution was evaluated by running the sample on the FemtoPulse system.

RNA was extracted from brain tissue of mChiNiv1 in the Tree of Life Laboratory at the WSI using the RNA Extraction: Automated MagMax™ *mir*Vana protocol ( do Amaral *et al.*, 2023). The RNA concentration was assessed using a Nanodrop spectrophotometer and a Qubit Fluorometer using the Qubit RNA Broad-Range Assay kit. Analysis of the integrity of the RNA was done using the Agilent RNA 6000 Pico Kit and Eukaryotic Total RNA assay.

### Hi-C sample preparation

Tissue from the muscle of the mChiNiv1 sample was processed for Hi-C sequencing at the WSI Scientific Operations core, using the Arima-HiC v2 kit. In brief, 20–50 mg of frozen tissue (stored at –80 °C) was fixed, and the DNA crosslinked using a TC buffer with 22% formaldehyde concentration. After crosslinking, the tissue was homogenised using the Diagnocine Power Masher-II and BioMasher-II tubes and pestles. Following the Arima-HiC v2 kit manufacturer's instructions, crosslinked DNA was digested using a restriction enzyme master mix. The 5’-overhangs were filled in and labelled with biotinylated nucleotides and proximally ligated. An overnight incubation was carried out for enzymes to digest remaining proteins and for crosslinks to reverse. A clean up was performed with SPRIselect beads prior to library preparation. Additionally, the biotinylation percentage was estimated using the Qubit Fluorometer v4.0 (Thermo Fisher Scientific) and Qubit HS Assay Kit and Arima-HiC v2 QC beads.

### Library preparation and sequencing

Library preparation and sequencing were performed at the WSI Scientific Operations core.

***PacBio HiFi***

At a minimum, samples were required to have an average fragment size exceeding 8 kb and a total mass over 400 ng to proceed to the low input SMRTbell Prep Kit 3.0 protocol (Pacific Biosciences, California, USA), depending on genome size and sequencing depth required. Libraries were prepared using the SMRTbell Prep Kit 3.0 (Pacific Biosciences, California, USA) as per the manufacturer's instructions. The kit includes the reagents required for end repair/A-tailing, adapter ligation, post-ligation SMRTbell bead cleanup, and nuclease treatment. Following the manufacturer’s instructions, size selection and clean up was carried out using diluted AMPure PB beads (Pacific Biosciences, California, USA). DNA concentration was quantified using the Qubit Fluorometer v4.0 (Thermo Fisher Scientific) with Qubit 1X dsDNA HS assay kit and the final library fragment size analysis was carried out using the Agilent Femto Pulse Automated Pulsed Field CE Instrument (Agilent Technologies) and gDNA 55kb BAC analysis kit.

Samples were sequenced using the Sequel IIe system (Pacific Biosciences, California, USA). The concentration of the library loaded onto the Sequel IIe was in the range 40–135 pM. The SMRT link software, a PacBio web-based end-to-end workflow manager, was used to set-up and monitor the run, as well as perform primary and secondary analysis of the data upon completion.

***Hi-C***

For Hi-C library preparation, DNA was fragmented using the Covaris E220 sonicator (Covaris) and size selected using SPRISelect beads to 400 to 600 bp. The DNA was then enriched using the Arima-HiC v2 kit Enrichment beads. Using the NEBNext Ultra II DNA Library Prep Kit (New England Biolabs) for end repair, A-tailing, and adapter ligation. This uses a custom protocol which resembles the standard NEBNext Ultra II DNA Library Prep protocol but where library preparation occurs while DNA is bound to the Enrichment beads. For library amplification, 10 to 16 PCR cycles were required, determined by the sample biotinylation percentage. The Hi-C sequencing was performed using paired-end sequencing with a read length of 150 bp on an Illumina NovaSeq 6000 instrument.

***RNA***

Poly(A) RNA-Seq libraries were constructed using the NEB Ultra II RNA Library Prep kit, following the manufacturer’s instructions. RNA sequencing was performed on the Illumina NovaSeq 6000 instrument.

### Genome assembly, curation and evaluation

***Assembly***

Prior to assembly of the PacBio HiFi reads, a database of *k*-mer counts ( *k* = 31) was generated from the filtered reads using FastK. GenomeScope2 ( Ranallo-Benavidez *et al.*, 2020) was used to analyse the *k*-mer frequency distributions, providing estimates of genome size, heterozygosity, and repeat content.

The HiFi reads were assembled using Hifiasm ( Cheng *et al.*, 2021) with the --primary option. Haplotypic duplications were identified and removed using purge_dups ( Guan *et al.*, 2020). The Hi-C reads were mapped to the primary contigs using bwa-mem2 ( Vasimuddin *et al.*, 2019). The contigs were further scaffolded using the provided Hi-C data ( Rao *et al.*, 2014) in YaHS ( Zhou *et al.*, 2023) using the --break option for handling potential misassemblies. The scaffolded assemblies were evaluated using Gfastats ( Formenti *et al.*, 2022), BUSCO ( Manni *et al.*, 2021) and MERQURY.FK ( Rhie *et al.*, 2020).

The mitochondrial genome was assembled using MitoHiFi ( Uliano-Silva *et al.*, 2023), which runs MitoFinder ( Allio *et al.*, 2020) and uses these annotations to select the final mitochondrial contig and to ensure the general quality of the sequence.

***Assembly curation***

The assembly was decontaminated using the Assembly Screen for Cobionts and Contaminants (ASCC) pipeline. Manual curation was conducted primarily in PretextView ( Harry, 2022) and HiGlass ( Kerpedjiev *et al.*, 2018), with additional insights provided by JBrowse2 ( Diesh *et al.*, 2023). Scaffolds were visually inspected and corrected as described by Howe *et al.* (2021). Any identified contamination, missed joins, and mis-joins were amended, and duplicate sequences were tagged and removed. Sex chromosomes were identified by read coverage analysis. The curation process is documented at https://gitlab.com/wtsi-grit/rapid-curation.

***Assembly quality assessment***

The Merqury.FK tool ( Rhie *et al.*, 2020), run in a Singularity container ( Kurtzer *et al.*, 2017), was used to evaluate *k*-mer completeness and assembly quality for the primary and alternate haplotypes using the *k*-mer databases ( *k* = 31) that were computed prior to genome assembly. The analysis outputs included assembly QV scores and completeness statistics.

A Hi-C contact map was produced for the final version of the assembly. The Hi-C reads were aligned using bwa-mem2 ( Vasimuddin *et al.*, 2019) and the alignment files were combined using SAMtools ( Danecek *et al.*, 2021). The Hi-C alignments were converted into a contact map using BEDTools ( Quinlan & Hall, 2010) and the Cooler tool suite ( Abdennur & Mirny, 2020). The contact map is visualised in HiGlass ( Kerpedjiev *et al.*, 2018).

The genome was also analysed within the BlobToolKit environment ( Challis *et al.*, 2020) and BUSCO scores ( Manni *et al.*, 2021) were calculated.

Table 4 contains a list of relevant software tool versions and sources.

**Table 4.** Software tools: versions and sources.

Software tool	Version	Source
BEDTools	2.30.0	https://github.com/arq5x/bedtools2
BLAST	2.14.0	ftp://ftp.ncbi.nlm.nih.gov/blast/executables/blast+/
BlobToolKit	4.2.1	https://github.com/blobtoolkit/blobtoolkit
BUSCO	5.3.2	https://gitlab.com/ezlab/busco
bwa-mem2	2.2.1	https://github.com/bwa-mem2/bwa-mem2
Cooler	0.8.11	https://github.com/open2c/cooler
fasta_windows	0.2.4	https://github.com/tolkit/fasta_windows
FastK	427104ea91c78c3b8b8b49f1a7d6bbeaa869ba1c	https://github.com/thegenemyers/FASTK
Gfastats	1.3.6	https://github.com/vgl-hub/gfastats
Hifiasm	0.16.1-r375	https://github.com/chhylp123/hifiasm
HiGlass	44086069ee7d4d3f6f3f0012569789ec138f42b84 aa44357826c0b6753eb28de	https://github.com/higlass/higlass
MerquryFK	d00d98157618f4e8d1a9190026b19b471055b22e	https://github.com/thegenemyers/MERQURY.FK
Minimap2	2.24-r1122	https://github.com/lh3/minimap2
MitoHiFi	2	https://github.com/marcelauliano/MitoHiFi
MultiQC	1.14, 1.17, and 1.18	https://github.com/MultiQC/MultiQC
PretextView	0.2.5	https://github.com/sanger-tol/PretextView
purge_dups	1.2.3	https://github.com/dfguan/purge_dups
samtools	1.19.2	https://github.com/samtools/samtools
sanger-tol/ascc	-	https://github.com/sanger-tol/ascc
Seqtk	1.3	https://github.com/lh3/seqtk
Singularity	3.9.0	https://github.com/sylabs/singularity
YaHS	1.2a	https://github.com/c-zhou/yahs

### Wellcome Sanger Institute – Legal and Governance

The materials that have contributed to this genome note have been supplied by a Darwin Tree of Life Partner. The submission of materials by a Darwin Tree of Life Partner is subject to the **‘Darwin Tree of Life Project Sampling Code of Practice’**, which can be found in full on the Darwin Tree of Life website here. By agreeing with and signing up to the Sampling Code of Practice, the Darwin Tree of Life Partner agrees they will meet the legal and ethical requirements and standards set out within this document in respect of all samples acquired for, and supplied to, the Darwin Tree of Life Project.

Further, the Wellcome Sanger Institute employs a process whereby due diligence is carried out proportionate to the nature of the materials themselves, and the circumstances under which they have been/are to be collected and provided for use. The purpose of this is to address and mitigate any potential legal and/or ethical implications of receipt and use of the materials as part of the research project, and to ensure that in doing so we align with best practice wherever possible. The overarching areas of consideration are:

•   Ethical review of provenance and sourcing of the material

•   Legality of collection, transfer and use (national and international)

Each transfer of samples is further undertaken according to a Research Collaboration Agreement or Material Transfer Agreement entered into by the Darwin Tree of Life Partner, Genome Research Limited (operating as the Wellcome Sanger Institute), and in some circumstances other Darwin Tree of Life collaborators.

## Data Availability

European Nucleotide Archive: *Chionomys nivalis* (European snow vole). Accession number PRJEB59810; https://identifiers.org/ena.embl/PRJEB59810. The genome sequence is released openly for reuse. The *Chionomys nivalis* genome sequencing initiative as part of the European Reference Genome Atlas Pilot Project ( https://www.erga-biodiversity.eu/pilot-project) and the Vertebrate Genomes Project (PRJNA489243). All raw sequence data and the assembly have been deposited in INSDC databases. The genome will be annotated using available RNA-Seq data and presented through the Ensembl pipeline at the European Bioinformatics Institute. Raw data and assembly accession identifiers are reported in Table 1 and Table 2.

## Author information

Members of the Wellcome Sanger Institute Tree of Life Management, Samples and Laboratory team are listed here: https://doi.org/10.5281/zenodo.12162482.

Members of Wellcome Sanger Institute Scientific Operations: Sequencing Operations are listed here: https://doi.org/10.5281/zenodo.12165051.

Members of the Wellcome Sanger Institute Tree of Life Core Informatics team are listed here: https://doi.org/10.5281/zenodo.12160324.

Members of the Tree of Life Core Informatics collective are listed here: https://doi.org/10.5281/zenodo.12205391.
