# Supplementary material for: The genome sequence of the European Snow Vole, Chionomys nivalis (Martins, 1842)
Source: Wellcome Open Res. 2025 Mar 20;10:156. [Version 1] doi: 10.12688/wellcomeopenres.23922.1 (PMC12531621; doi:10.12688/wellcomeopenres.23922.1)
Supplement: Peer review history

# Reviewer report: Reviewer response for version 1

**DOI:** 10.21956/wellcomeopenres.26391.r134368

**Published:** 2025-10-16

**Authors:** Nathanael Herrera

**Affiliations:** 1 University of Montana, Missoula, Montana, USA

This genome note reports a new chromosome-level assembly for the European snow vole (Chionomys nivalis). The authors present a 2.39 Gb genome generated with PacBio HiFi long reads and Hi-C scaffolding, achieving an N50 of 88.84 Mb and 96.2% BUSCO completeness. The assembly also includes the complete mitochondrial genome. The methods follow current best practices for genome assembly, resulting in a high-quality reference.

 The manuscript is transparent and detailed, though the RNA-seq component needs clearer explanation. Several tissues were collected, but it is unclear which ones (beyond brain) were sequenced for the RNA-seq dataset. As noted by another reviewer, the results and additional details of this component should be included.

 Overall, this is a well-executed study that provides a valuable genomic resource for *C. nivalis*.

Are sufficient details of methods and materials provided to allow replication by others?

Yes

Is the rationale for creating the dataset(s) clearly described?

Yes

Are the datasets clearly presented in a useable and accessible format?

Yes

Are the protocols appropriate and is the work technically sound?

Yes

Reviewer Expertise:

evolutionary genetics, bioinformatics, Mammalogy

I confirm that I have read this submission and believe that I have an appropriate level of expertise to confirm that it is of an acceptable scientific standard.

# Reviewer report: Reviewer response for version 1

**DOI:** 10.21956/wellcomeopenres.26391.r121140

**Published:** 2025-04-05

**Authors:** Naoki Osada

**Affiliations:** 1 Hokkaido University, Sapporo, Japan

The authors performed a genome assembly of the European snow vole ( *Chionomys nivalis*) using long-read and Hi-C sequencing data. The assembly process followed standardized methods, and the resulting assembly appears to be of reasonable quality. I have only three minor suggestions to help complete the manuscript:

 1. Has any karyotype data for *C. nivalis* been reported in previous studies? If so, referencing this would help support the validity of the genome assembly results.

 2. Although metadata for the sequenced individual is presented in the Methods section, it would be helpful to also include this information in Table 1.

 3. RNA-seq data were used for gene annotation, but this is only mentioned in the Methods section. A brief summary of the RNA-seq results, such as the number of predicted genes, should be included in the Results section for completeness.

Are sufficient details of methods and materials provided to allow replication by others?

Yes

Is the rationale for creating the dataset(s) clearly described?

Yes

Are the datasets clearly presented in a useable and accessible format?

Yes

Are the protocols appropriate and is the work technically sound?

Yes

Reviewer Expertise:

population genetics, molecular evolution, genomics

I confirm that I have read this submission and believe that I have an appropriate level of expertise to confirm that it is of an acceptable scientific standard.
